# Death from Ether

**Published:** 1873-02

**Authors:** W. B. Dunning

**Affiliations:** Acting House Surgeon, Bellevue Hospital.


### ARTICLE VII.

### *Death from Ether.*

BY W. B. DUNNING, M. D.

Acting House Surgeon, Bellevue Hospital.

John Stockander, a German saddler, unmarried, 68 years old, was admitted to ward 13 of Bellevue Hospital on August 2, 1872, suffering from a fracture of left femur, just below the trochanter. The patient was treated by a Buck's Extension until August 20, when it was decided to apply a plaster of Paris splint. In order to make sufficient extension, and at the same time prevent the pain of the operation, ether was ordered to be administered. The administration of the anæsthetic was slowly and carefully made, and after perhaps ten minutes the patient was fully under its influence and the operation begun. A few turns of the plaster had been made, when the patient's breathing was observed to be rather frequent and gasping. The pulse was, however full and regular. The thorax was compressed two or three times, and the patient's breathing again became normal. As these symptoms do not rarely occur during etherization, they excited no special alarm. The ether was, however, withheld from the patient four or five minutes, his respiration and pulse being normal. As he then began, however, to move about and his muscles were becoming rigid, the ether cone was again applied. In a minute or two, my assistant, who was giving the ether, observed the pupils to be dilating rapidly and the breathing to cease. His heart was still beating, however. The ether cone was of course immediately removed and artificial respiration was again used, and all the batteries obtainable in the hospital were put in operation in an effort to resuscitate the patient. His muscles occasionally responded by a spasmodic movement, but no breathing again occurred. The efforts of resuscitation were continued about forty minutes, until all response to the action of the battery had ceased.

Patient died about four P. M. and the autopsy was made at seven P. M. that same day, under direction of Dr. Delafield. Rigor mortis was marked. Blood was fluid. Brain and membranes neither anæmic nor congested. Trachea and larynx somewhat pale. Heart contained a little fluid blood, with a little atheroma at base of aortic valves. Lungs have old adhesions over both. Emphysema exists, and thickening of large bronchi. The lower lobe of right lung is œdematus, and its lower portion in a state of red hepatization. Rest of lung is normal and not congested. Liver is small and firm, containing a good deal of fluid blood. The other organs are normal.

The ether used was that made by Powers & Weightman, It has been examined by Dr. Squibb, of Brooklyn. He states that he " finds nothing in the character or quality of the ether to account for the death of the man, or even to aid in accounting for it." He adds, that in his "judgment the death of this patient is in no way attributable to either the quality of the ether, the quantity used, or the mode of administration, but that it is one of those accidents which, though of very rare occurrence under the careful use of ether, is inseparable from the condition of anæsthesia."

The quantity of ether used was about ℥ vi.—*Medical Record.*

